# Non-adherence to WHO’s recommended 8-contact model: geospatial analysis of the 2017 Maternal Health Survey

**DOI:** 10.1186/s12884-023-05504-w

**Published:** 2023-03-18

**Authors:** Kwamena Sekyi Dickson, Ebenezer N. K. Boateng, Kenneth Setorwu Adde, Edward Kwabena Ameyaw, Michelle L. Munro-Kramer

**Affiliations:** 1 Department of Population and Health, University of Cape Coast, Cape Coast, Ghana; 2 Department of Geography and Regional Planning, University of Cape Coast, Cape Coast, Ghana; 3 Institute of Policy Studies and School of Graduate Studies, Lingnan University, Hong Kong, Hong Kong; 4 L and E Research Consult Ltd, Upper West Region Wa, Ghana; 5 University of Michigan School of Nursing, Ann Arbor, USA

**Keywords:** Antenatal care, Reproductive health, Women, Geospatial analysis, Ghana, Social demography

## Abstract

**Introduction:**

Evidence shows that most women in Ghana do not meet the minimum 8-contact model for antenatal care as recommended by WHO with only 31.2%-41.9% of them meeting the recommendation. To the best of our knowledge, no study in Ghana has examined women’s noncompliance with the WHO’s recommended 8-contact model for antenatal care using geospatial analysis, as this study sets out to do.

**Methods:**

We sourced data from the recent version of the Ghana Maternal Health Survey which was executed in 2017. A sample of 10,077 women with complete data participated in this study. The link between the explanatory variables and the outcome variable was investigated using binary and multivariate logistic regression models and Spatial analyses such as spatial autocorrelation (Moran's I), hotspot, cluster and outlier analysis, and geographically weighted regression were conducted using ArcMap version 10.7.

**Results:**

Districts found in the north-eastern and south-western parts of the country were more likely to experience noncompliance with ANC. Women staying within the middle belt without health insurance were more likely (17–29%) to be noncompliant with ANC. Women with low community socioeconomic status were found to be more likely (17–34%) to be noncompliant with ANC in the eastern parts of Ghana.

**Conclusion:**

The study has shown that in order to achieve targets one and three of Sustainable Development Goal 3, the government of Ghana, the Ministry of Health, together with the Ghana Health Service may have to intensify health education in the identified areas to highlight the importance of adherence to the WHO recommendations on ANC 8-contact model.

**Supplementary Information:**

The online version contains supplementary material available at 10.1186/s12884-023-05504-w.

## Introduction

For better pregnancy outcomes, effective use of antenatal care (ANC) services is vital [1–3], however, it is estimated that about five out of ten women do not receive adequate ANC services in low-and middle-income countries (LMIC) [2, 4]. Nonetheless, ANC utilisation over the years has increased even in LMIC countries, however, this increase does not reflect in a reduced maternal mortality or morbidity [5–7]. Hence the need for a paradigm shift from ANC utilisation to quality ANC utilisation [8].

In view of this, the World Health Organisation (WHO) in 2016 came up with recommendations to increase ANC visits from four to eight [8]. This has proven to be effective from studies that have shown that more ANC visits lower the chances of stillbirths and perinatal mortality [9, 10]. This has also helped in the early detection and prevention of adverse pregnancy outcomes [10–12], infant mortality and morbidity [13–15]. ANC has the potency to help attain the SDG3 target 2 which seeks to end all preventable death of new-borns and children under 5 years.

In Ghana, a free maternal healthcare policy was introduced in the year 2008 to improve ANC utilisation, skilled birth delivery, and postnatal care service utilisation [16, 17]. This has reflected positively in the number of women who make at least one ANC visit (98 percent) and four or more (90 percent) with skilled health professionals [18]. However, with regards to the WHO’s recommended 8-contact model, Kumbeni and colleagues [19] recorded 31.2 percent, whiles Ekholuenetale, Nzoputam and Barrow [20] on the other hand recorded 41.9 percent. These evidences indicate relatively lower prevalence with respect to the recommended 8-contact model.

Besides, several studies have attempted to examine the factors that influence the utilisation of ANC in Ghana. For instance, Ekholuenetale, Nzoputam and Barrow [20] investigated the prevalence and socio-economic differences of eight or more ANC contacts and observed that the recommended eight or more ANC contacts recommended by WHO in 2016 is yet to be fully institutionalised. Afulani [13] on the other hand found that good quality ANC can improve the birth outcomes in Ghana and also promote health facility delivery. Afaya et al. [21] assessed the factors associated with optimum utilisation of ANC in rural Ghana and observed that although awareness of ANC is high, there is a need for further education to increase utilisation. Kumbeni et al. [19] also argue that spending at least 20 min during the first ANC contact and home visit has a positive association with eight or more ANC contacts. However, to the best of our knowledge, none of the previous studies examined women’s noncompliance with the WHO’s recommended eight ANC contacts using geospatial analysis. This notwithstanding the fact that geospatial analysis helps in identifying at-risk populations and targeting interventions [22, 23]. By incorporating location data and mapping, geospatial analysis enables the examination of the relationship between noncompliance with ANC and various contextual factors, such as demographic characteristics, access to healthcare facilities, poverty levels, and cultural attitudes towards prenatal care. There is therefore a need to investigate the geospatial differentials in Ghana regarding noncompliance with ANC utilisation. Findings from this study will enable the government and policymakers of Ghana to implement policies that will target the needs of each geographic area to improve compliance with quality ANC.

## Methods

### Data source

This study used the most recent Ghana Maternal Health Survey (GMHS) data. The 2017 GMHS used a standard model questionnaire developed by the Measure DHS Programme. The 2017 GMHS is a national representative household survey that collect comprehensive information on maternal health issues- antenatal care, delivery, postnatal care, maternal mortality and child mortality. The 2017 GMHS interviewed 25,062 women from 26,324 households within 898 clusters in 1900 enumeration areas [18]. For the study, women with birth history who had given birth up to five years before the survey were included. A sample of 10,077 women with complete data required for our analysis participated in this study. Permission to use the data set was given by the MEASURE DHS following the assessment of our concept note. The datasets are freely available to the public at www.measuredhs.com.

### Definition of variables and measurement

#### Outcome variable

The outcome variable was ANC noncompliance. This was categorized as follows: (0) compliance with WHO’s recommended ANC 8-contact model (eight or more ANC visits) and (1) noncompliance with WHO’s recommended ANC 8-contact model (less than eight ANC visits). This conceptualisation was premised on the new recommended guidelines by the WHO were considered [8], making less than eight visits equivalent to noncompliance or inadequate or insufficient ANC visits.

#### Explanatory variables

Individual Level Explanatory Variables included age, wealth status was derived from household ownership of a diversity of assets and categorized as poorest, poorer, middle, richer, richest, marital status, educational status, household size, head of household sex, parity, and health insurance. Mass media exposure was computed from frequency of listening to radio, frequency of reading newspapers and magazine, and the frequency of watching television. Those who were exposure to mass media at least one was captured as “yes” and those who were not exposed were captured as “no”.

Community Level Explanatory Variables. Community level factors included and coded as follows: distance to health facility (big problem and not a big problem); community socioeconomic status (low, medium, and high); place of residence (urban and rural); and community literacy level (low, medium, and high). The distance to health facility was coded as a big problem if a pregnant woman reported that the distance to a health centre or hospital was a big problem for her. Community socioeconomic status was computed from the occupation, wealth, and education of study participants who resided in a given community. Principal component analyses were applied to calculate women who were unemployed, uneducated, and poor. A standardized rating was derived with an average rating (zero) and standard deviation [3, 9]. The rankings were then segregated into tertile 1 (least disadvantaged), tertile 2, and tertile 3 (most disadvantaged) where the least rating (tertile 1) denoted greater socioeconomic status with the highest score (tertile 3) denoting lower socioeconomic status. Places of residence were coded as urban and rural.

Correspondingly, for community literacy, respondents who had attended higher than secondary school were assumed to be literate while all other respondents were given a sentence to read, and they were considered literate if they could read all or part of the sentence. Therefore, high literacy included respondents who had higher than secondary education or had no school/primary/secondary education and could read a whole sentence. Medium literacy means respondents who had no school/primary/secondary education and could read part of the sentence. Low literacy means respondents who had no school/primary/secondary education and could not read at all. These were categorized into appropriate tertiles where tertile 1 (lowest score and least disadvantaged) was high community literacy, tertile 2 (medium score) was medium community literacy, and tertile 3 (highest score and most disadvantaged) was low community literacy.

## Data analysis

We used descriptive and inferential statistics. Percentages were used to report descriptive data. The link between the explanatory variables and the outcome variables was investigated using binary and multivariate logistic regression models. The survey command in Stata was used to correct for the complicated sample structure of the data in the regression analysis, while all frequency distributions were weighted. The odds ratios (ORs) with 95 percent confidence intervals were used to present the results of the logistic analyses (CIs).

### Model fit and specifications

The Likelihood Ratio (LR) test was used to evaluate the fitness of all of the models. Before fitting the models, the presence of multicollinearity between the independent variables was evaluated. The variance inflation factor (VIF) test found that the variables were not highly multicollinear (Mean VIF = 1.98).

### Spatial analysis

Regarding the spatial analysis, the coordinates gathered during the data collection was downloaded from the DHS website (http://www.measuredhs.com/). Spatial analysis is a way of visualising locations of women in the study. The coordinates were projected from geographic coordinate (WGS 1984) to projected coordinate (Ghana Meter Grid). Since the focus of the study was at the district level, the district shapefile of Ghana (216 districts) was obtained from the Department of Geography and Regional Planning, University of Cape Coast, for the analysis. Information from the district shapefile, such as district names, were merged with the surveyed coordinates. This action aims to tie the district information to the respondents surveyed in this study. It is worth mentioning that not all districts had respondents sampled for the survey. Such districts were excluded from the dataset. The extracted GMHS data were merged with coordinates using the cluster information. The extracted data had ANC (0 = compliance and 1 = noncompliance) and the explanatory variables were estimated in proportions with a focus on the least category. For instance, proportions were created for those with no National Health Insurance Scheme (NHIS), respondents aged 15–19, residents in rural areas and others. However, since the cluster information has been tied to the district names, it made it easier for spatial join. The spatial join was transferred to the extracted data to the 216-district shapefile using ArcMap version 10.7. It was observed that some of the districts had more than one cluster and in such cases, the data from the clusters were aggregated. Thus, their means (averages) were computed to represent their respective districts [22, 23].

After the data management, the first level of analysis was to assess the spatial distribution of ANC in Ghana using the Spatial Autocorrelation (Global Moran’s I) tool in ArcGIS version 10.7. This was based on the hypothesis that the occurrence of noncompliance to ANC is random. The results (Additional file 1: Appendix A) showed that the distribution of ANC attendance in Ghana was clustered, rejecting the null hypothesis. This necessitated conducting further analysis such as the Hotspot analysis (Getis-Ord G), Cluster and Outlier analysis and Geographically Weighted Regression (GWR) analysis. It is worth noting that the Spatial Autocorrelation (Moran’s I) results do not show the exact districts with a higher incidence of noncompliance of ANC. Therefore, the Hotspot analysis (Getis-Ord G) was deemed appropriate to show districts with a higher incidence of noncompliance of ANC. Although the Hotspot analysis (Getis-Ord G) shows hot and cold spot areas of the incidence of ANC noncompliance, there is a likelihood of overgeneralisation that may not support target-specific policies and interventions. This led to the Cluster and Outlier analysis, which reveals findings that may be overgeneralised by the Hotspot analysis. Thus, this analysis reveals outlier districts be it hot or cold spots not captured in the Hotspot results. The GWR was conducted to understand the spatial predictor of noncompliance to ANC in Ghana. The GWR is a spatial regression modelling tool used to determine specific explanatory variables that best account for the observed spatial patterns of noncompliance of ANC in Ghana [22, 24]. Prior to running the GWR analysis, the Ordinary Least Square (OLS) analysis was conducted to identify significant spatial predictors of ANC noncompliance (Additional file 1: Appendix B). This approach is a prerequisite to running the GWR. The OLS results identified five (5) predictors of noncompliance of ANC. The predictors were used for the GWR analysis, and the outputs are presented in maps.

## Results

### Background characteristics and proportion of ANC noncompliance

A higher proportion of the respondents (75.7%) aged 15–19 are not ANC compliant. Women with poorest wealth status (73.2%), no education (67.5%), never in union (65.7%), with a 5 + household size (61.1%), with parity of 5 + (62.4%), no health insurance coverage (57.4%) and no media exposure (74.0%) were not ANC compliant (see Table 1). Women from communities with low socioeconomic status (66.8%) and low literacy level (70.2%) also recorded a higher rate of noncompliance. A higher proportion of women from rural residence (62.6%) and those who saw distance to health facility as a big problem (62.7%) were not compliant with their ANC utilization (see Table 1).

**Table 1:** Background characteristics

Variable	Frequency (*n* = 10,077)	Proportion of Noncompliance (less than 8 Contacts)	95% Confidence interval
**Individual Variables**			
*Age*			
15–19	509	75.7	74.4–76.9
20–24	1,802	63.1	62.4–63.9
25–29	2,471	52.8	52.1–53-4
30–34	2,431	49.8	49.1–50.5
35–39	1,780	50.7	50.0–51.4
40–44	808	54.6	53.4–55.7
45–49	276	56.1	54.2–58.0
***Wealth status***			
Poorest	2,003	73.2	72.6–73.8
Poorer	2,134	65.8	65.1–66.5
Middle	2,013	56.4	55.7–57.1
Richer	2,065	46.3	45.6–47.0
Richest	1,962	30.9	30.3–31.6
***Educational status***			
No education	2,289	67.5	66.9–68.1
Primary	1,685	62.5	61.8–63.3
Secondary	5,421	51.0	50.6–51.4
Higher	682	25.5	24.4–26.6
***Marital status***			
Never in union	898	65.7	64.6–66.7
Married	5,479	51.9	51.4–52.2
Living with partner	3,108	57.2	56.6–57.7
Widowed	119	61.1	58.2–63.9
Divorced	108	44.4	58.2–63.9
Separated	365	57.2	55.5–58.8
***Household size***			
< 5	5,840	50.5	50.0–50.9
5 +	4,237	61.1	60.6–61.6
***Sex of household head***			
Male	7,246	55.1	54.7–55.4
Female	2,831	54.6	54.0–55.2
***Parity***			
1–2	4,730	52.0	51.5–52.4
3–4	3,118	54.2	53.6–54.7
5 +	2,229	62.4	61.7–63.1
***Health insurance coverage***			
No	4,234	57.4	56.9–57.9
Yes	5,843	53.2	52.8–53.6
***Mass media exposure***			
No	1,029	74.0	73.1–74.9
Yes	9,048	52.8	52.4–53.1
**Community variables**			
***Community socioeconomic status***			
Low	4,821	66.8	66.4–67.2
Moderate	922	55.8	54.7–56.8
High	4,334	41.6	41.1–42.1
***Community literacy level***			
Low	2,309	70.2	69.5–70.8
Moderate	3,613	56.7	56.2–57.3
High	4,155	45.0	44.5–45.5
***Place of residence***			
Urban	5,002	47.2	46.8–47.7
Rural	5,075	62.6	62.1–63.0
***Distance to health facility***			
Big problem	2,473	62.7	62.0–63.3
Not a big problem	7,604	52.4	52.1–52.8
Total	**10,077**	55.0	54.6–55.3

Source: Computed from 2017 Maternal Health Survey

### Logistic regression of ANC noncompliance among Ghanaian women

Age, wealth status, educational level, marital status, parity, mass media, community socioeconomic status and place of residence were seen to have a significant relationship with ANC noncompliance among Ghanaian women.

The likelihood of ANC noncompliance varied with age. For instance, younger women aged 15 – 19 years (OR = 1.96, CI = 1.53, 2.51) had a higher likelihood of ANC noncompliance compared to the reference category of those 25 – 29 years. Whereas older women aged 45 – 49 years (OR = 0.68, CI = 0.52, 0.89) had a lesser likelihood of ANC noncompliance compared to the reference category of those 25 – 29 years. Women with richest wealth status (OR = 0.42, CI = 0.34, 0.52) had lesser likelihood of ANC noncompliance compared to those with poorest wealth status (see Table 2).

**Table 2:** Logistic regression of ANC noncompliance among Ghanaian women

**Variable**	**Odds Ratio**	**95% Confidence Interval**	
		**Lower bound**	**Upper bound**
*Age*			
15–19	1.96***	1.53	2.51
20–24	1.37***	1.19	1.57
25–29	Ref	Ref	Ref
30–34	0.86**	0.76	0.98
35–39	0.75***	0.65	0.88
40–44	0.80**	0.66	0.97
45–49	0.68**	0.52	0.89
***Wealth status***			
Poorest	Ref	Ref	Ref
Poorer	0.92	0.81	1.06
Middle	0.75***	0.64	0.88
Richer	0.59***	0.49	0.71
Richest	0.42***	0.34	0.52
***Educational status***			
No education	1.32***	1.16	1.49
Primary	1.09	0.96	1.23
Secondary	Ref	Ref	Ref
Higher	0.92	0.75	1.11
***Marital status***			
Never in union	1.51***	1.24	1.83
Married	Ref	Ref	Ref
Living with partner	1.11	0.99	1.24
Widowed	1.29	0.87	1.92
Divorced	0.68	0.44	1.07
Separated	1.18	0.91	1.55
***Household size***			
< 5	Ref	Ref	Ref
5 +	1.09	0.99	1.19
***Sex of household head***			
Male	Ref	Ref	Ref
Female	0.94	0.84	1.04
***Parity***			
1–2	Ref	Ref	Ref
3–4	1.35***	1.19	1.52
5 +	1.49***	1.26	1.76
***Health insurance coverage***			
No	1.03	0.94	1.12
Yes	Ref	Ref	Ref
***Mass media exposure***			
No	1.33***	1.17	1.53
Yes	Ref	Ref	Ref
**Community variables**			
***Community socioeconomic status***			
Low	Ref	Ref	Ref
Moderate	0.91	0.77	1.08
High	0.68***	0.60	0.78
***Community literacy level***			
Low	1.10	0.98	1.24
Moderate	Ref	Ref	Ref
High	0.99	0.89	1.12
***Place of residence***			
Urban	1.13*	1.01	1.08
Rural	Ref	Ref	Ref
***Distance to health facility***			
Big problem	1.11	1.00	1.22
Not a big problem	Ref	Ref	Ref

Source: Computed from 2017 Maternal Health Survey *p < 0.05 **p < 0.01 ***p < 0.001

Ref: Reference Category

Women who had never been in union (OR = 1.51, CI = 0.24, 1.83) had a higher likelihood of ANC noncompliance compared to married women. The odds of ANC noncompliance were higher for women with no formal education (OR = 1.32, CI = 1.16, 1.49) and women with 5 + parity (OR = 1.49, CI = 1.26, 1.76) compared to women with secondary education and those with parity 1–2. Women who had no mass media exposure (OR = 1.33, CI = 1.17, 1.53) had a higher likelihood of ANC noncompliance compared to women with mass media exposure (see Table 2).

### Spatial distribution of ANC noncompliance

Results from the spatial autocorrelation analysis revealed that ANC’s spatial distribution of noncompliance in Ghana is not randomised but clustered (Additional file 1: Appendix A). This shows that the distribution of noncompliance of ANC occurs in some specific areas in Ghana. Since the spatial autocorrelation results do not show the specific areas where the noncompliance of ANC occurs. The hotspot analysis was conducted to identify the specific districts in Ghana noncompliance of ANC occurs.

**Fig. 1:**
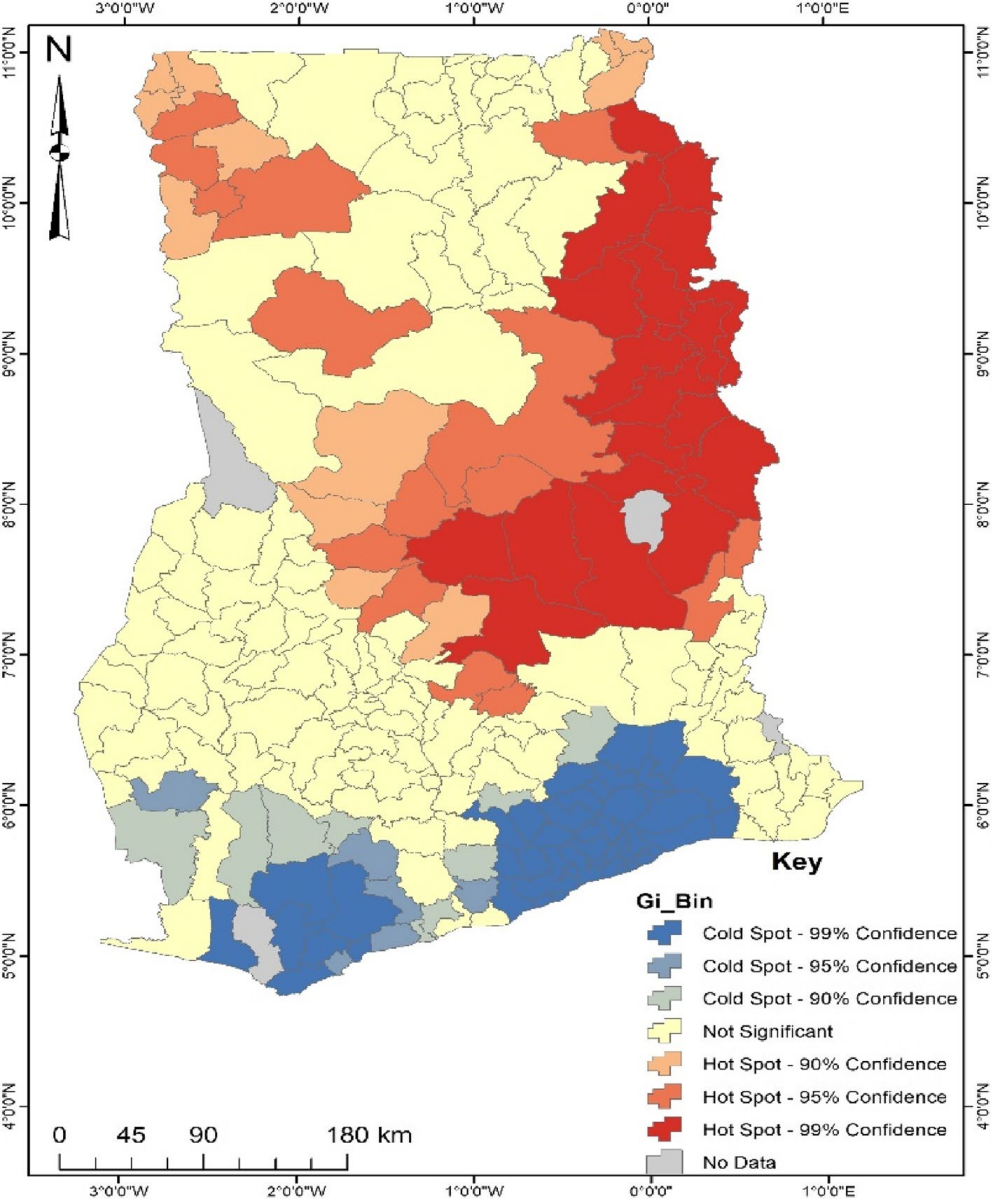
Hotspot analysis of ANC. Source: Computed from 2017 Maternal Health Survey

According to Fig. 1, it was found that the hotspot for the noncompliance of ANC predominantly occurs in north-eastern Ghana, with a few occurring in the north-western. Some districts identified to be the hotspot for noncompliance of ANC are Atebubu Amantin, Sekyere Afram Plain North, Sene West, Sene East, Krachi East, Krachi Nchumuru, Nkwanta South, Nkwanta North, Kpandi, Nanumba South, Nanumba North, Mion, Yendi Municipal, Tatale, Zabzugu, Saboba, Gushiegu, Chereponi, Bunkpurugu Yonyo, West Gonja and Wa West. This means that there is a high tendency of women engaging in noncompliance of ANC in the districts mentioned above. Areas in the southern part of Ghana were found to be cold spots for noncompliance of ANC. Some districts identified as cold spots are Ga South, Nsawam Adoagyiri, Accra Metropolis, Tema Metropolis, Tarkwa Nsuaem, Ahanta West, Sekondi Takoradi Metropolis and Komenda Edna Eguafo Abirem. This implies that most women in such districts are more likely to attend ANC.

**Fig. 2:**
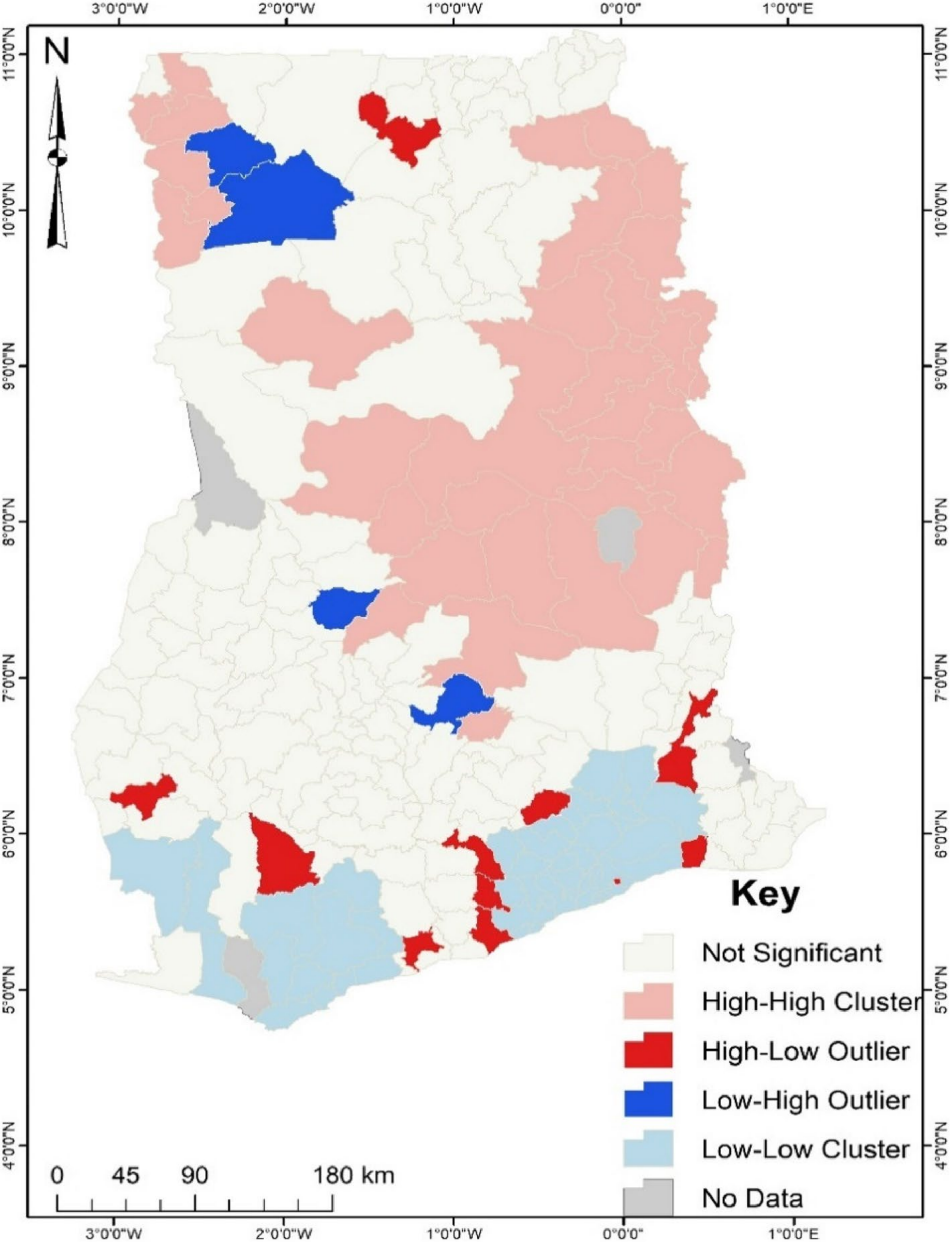
Cluster and Outlier of ANC. Source: Computed from 2017 Maternal Health Survey

Although the hotspot analysis gives a spatial view of the areas with a high proportion of noncompliance of ANC, the cluster and outlier analysis (Fig. 2) revealed some unique findings that were grossed over by the hotspot analysis. For instance, a district such as Wa West was found to be a hotspot for noncompliance of ANC. However, the cluster and outlier results (Fig. 2) showed that the district has a low proportion of noncompliance of ANC but is surrounded by a district with a high incidence of ANC noncompliance. Other districts with a low occurrence of ANC noncompliance were Dffiama Bussie, Nkoranza North and Asante Akim North.

It was also found that districts such as Wassa Amenfi West, Bodi, Abura Asebu Kwamankese, Builsa South, Birim Municipal, Agona West, Gomoa West, East Akim, Ashaiman, Ada West and Ho West were found to have a high incidence of noncompliance of ANC but were surrounded by districts with low incidence. The hotspot analysis could not capture such areas. The other districts identified having a high and low incidence of noncompliance of ANC and surrounded by districts with similar occurrence corresponded to the findings of the hotspot analysis.

### Geographic Weighted Regression of noncompliance of ANC

In addition, the OLS analysis was conducted to identify the significant spatial predictors of noncompliance of ANC in Ghana using proportions of the explanatory variable. The results from the OLS analysis (Additional file 1: Appendix B) revealed that five (5) factors significantly account for the spatial distribution of the noncompliance of ANC in Ghana. These five (5) factors were household size, marital status, National Health Insurance Scheme, community socioeconomic status and community literacy status. These factors were then used for the GWR analysis.

GWR is a local model that estimates the different responses of the dependent variable on independent variables based on locational effects [22]. This explains the spatial predictive power of the significant factors explaining the occurrence of the noncompliance of ANC in Ghana. The GWR model fit parameters were; Sigma- 0.142, AICc- 204.778, R^2^- 0.499 and R^2^ adjusted- 0.410. Deducing from the Adjusted R^2^, the GWR explains about 41% of the dataset. Areas depicting red on the maps generated from the GWR explain a strong predictive power of the determinants of noncompliance of ANC, whereas blue shows a weak predictive power.

**Fig. 3:**
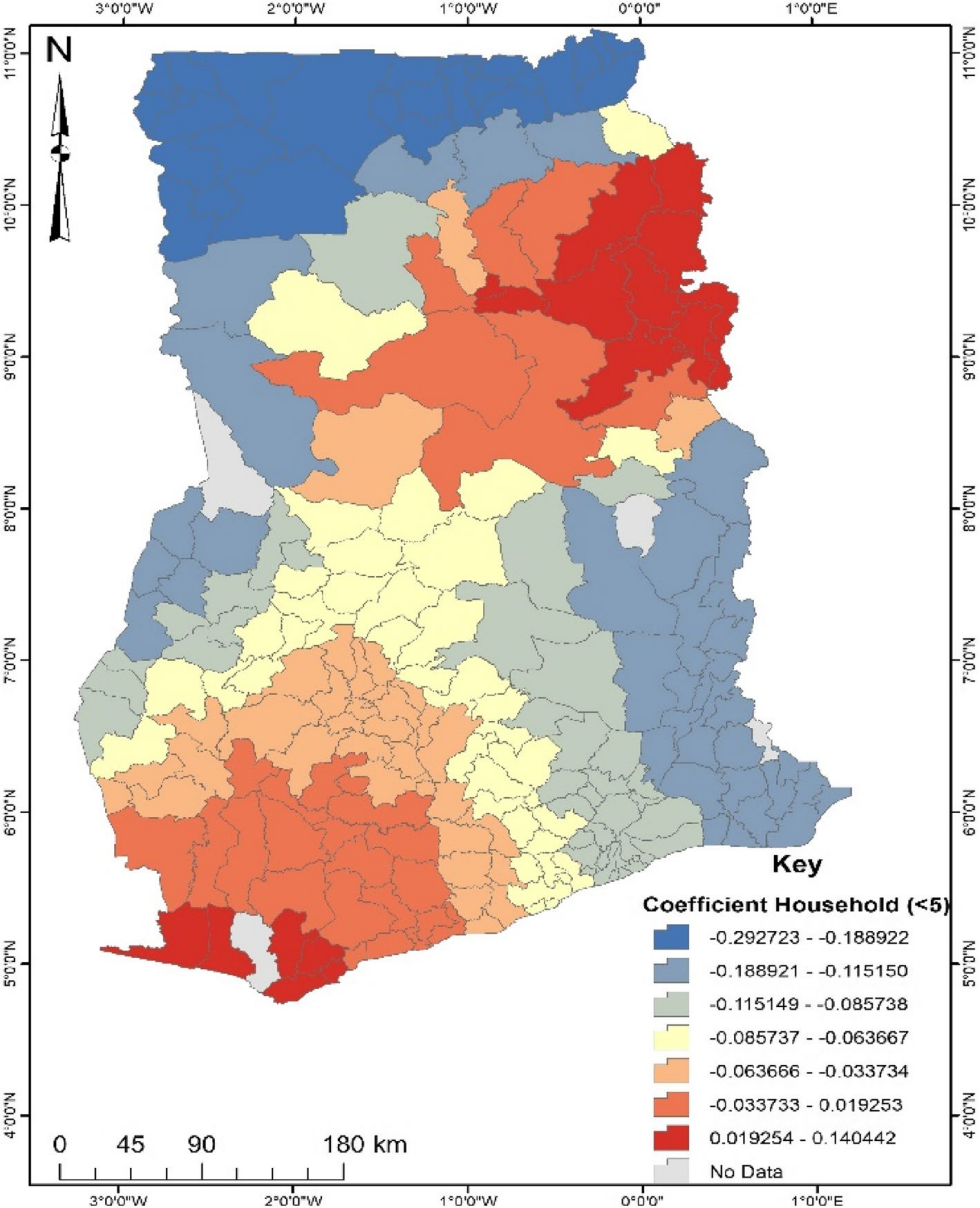
Coefficient of Household size less than 5. Source: Computed from 2017 Maternal Health Survey

According to Fig. 3, districts located in the north-eastern and south-western districts are more likely to experience noncompliance of ANC when households are less than five (5). This implies that in such areas women who have a household size of less than five (5) stand a higher chance (1–14%) of engaging in the noncompliance of ANC. The vice-versa was observed for women who reside in the northern and south-east of Ghana.

**Fig. 4:**
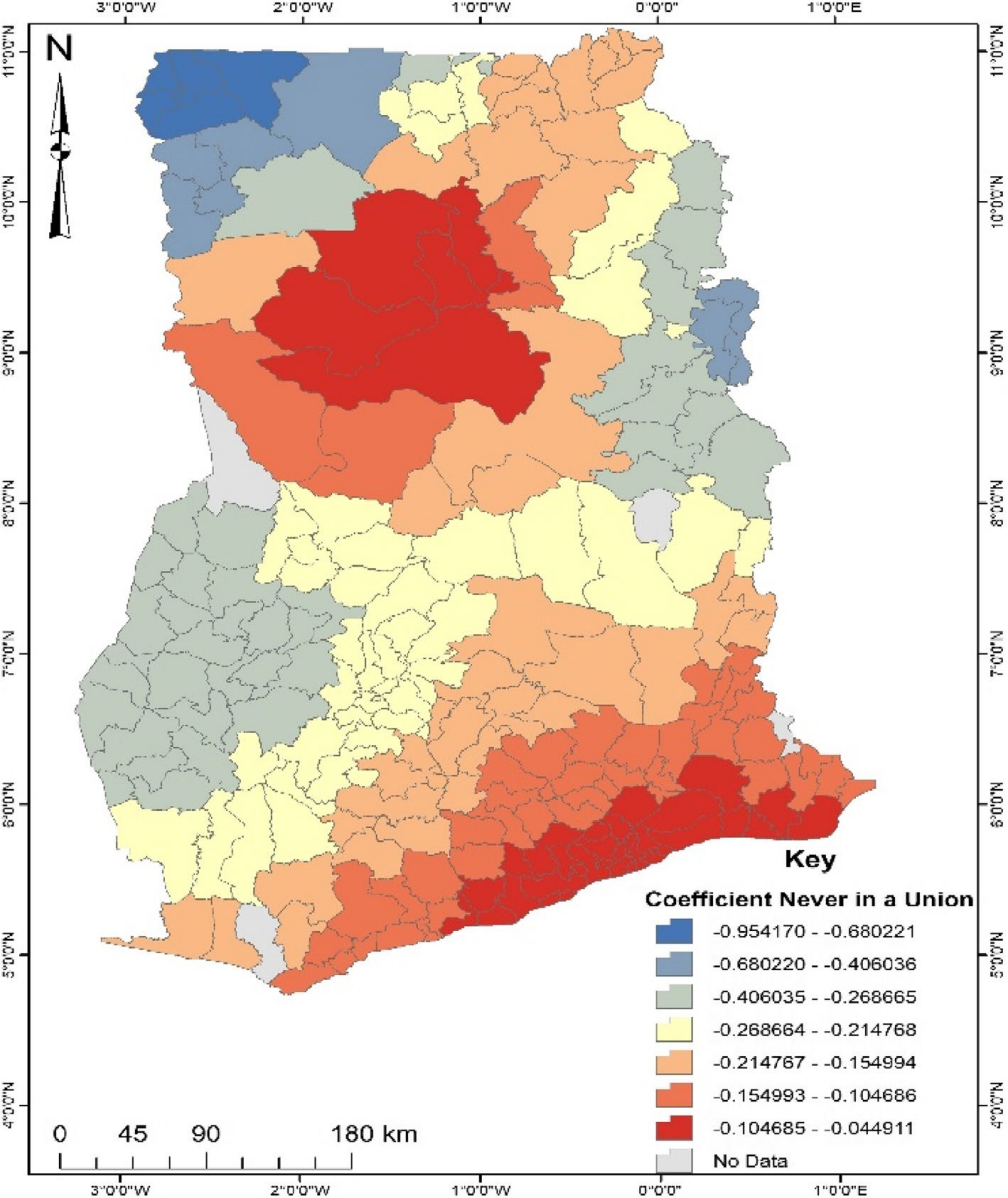
Coefficient of Marital status never in a union. Source: Computed from 2017 Maternal Health Survey

Regarding marital status (Fig. 4), proportions of women never in a union are likely (4–10%) not to engage in ANC in the south-eastern and some parts of the north. Thus, women who have never been in a union living in the south-eastern and some parts of the north in Ghana tend to engage in noncompliance with ANC. On the other hand, women in the north-west and some parts of the north-east who are never in a union are less likely (40–95%) to engage in noncompliance with ANC.

**Fig. 5:**
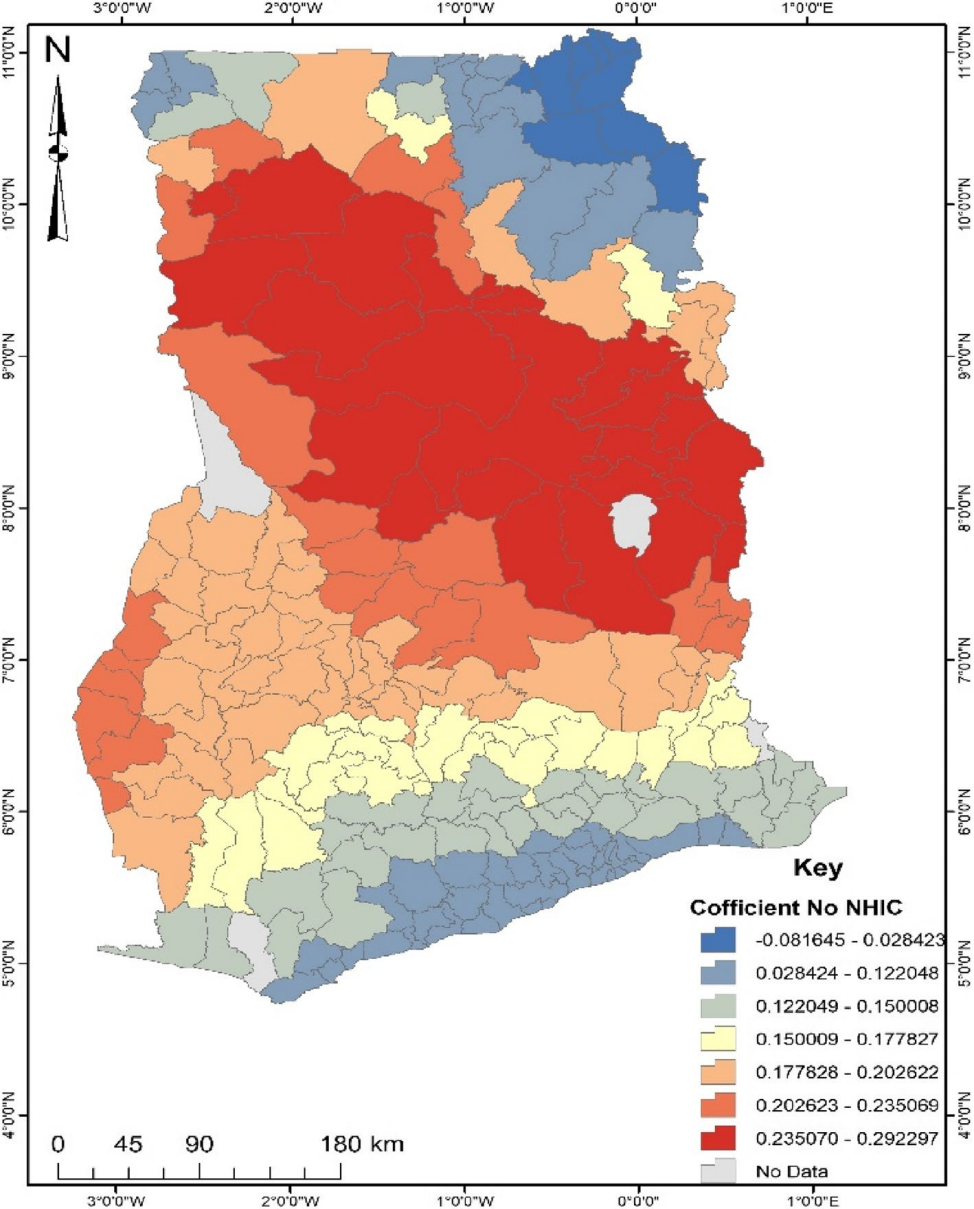
Coefficient of No NHIS. Source: Computed from 2017 Maternal Health Survey

According to Fig. 5, women staying within the country’s middle belt without health insurance were more likely (17–29%) to be non-compliant with ANC. Thus, the noncompliance of ANC by women in the middle of Ghana is due to non-subscription to the National Health Insurance Scheme. Other women in the southern and north-eastern part of Ghana are less likely (6–12%) to be non-compliant of ANC regardless of NHIS.

**Fig. 6:**
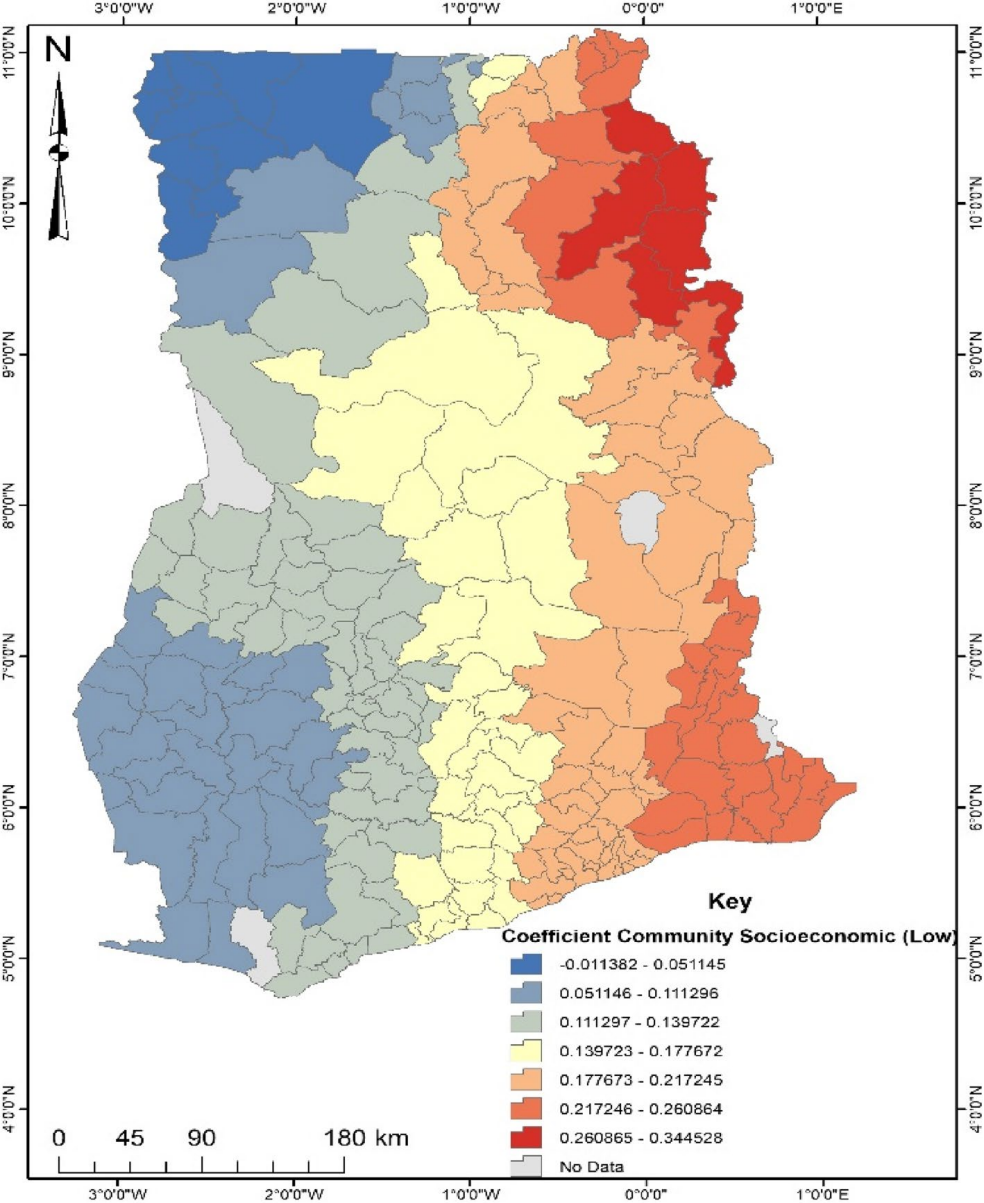
Coefficient of Community Socioeconomic (Low). Source: Computed from 2017 Maternal Health Survey

From Fig. 6, women with low community socioeconomic status were found to be more likely (17–34%) to be non-compliant with ANC in the eastern parts of Ghana. This implies that women in the eastern part of the country with low community socioeconomic status have a higher tendency to engage in ANC noncompliance. Contrary, women found in the north-west and south-west are less likely (1–11%) to engage in noncompliance of ANC even with low community socioeconomic status.

**Fig. 7:**
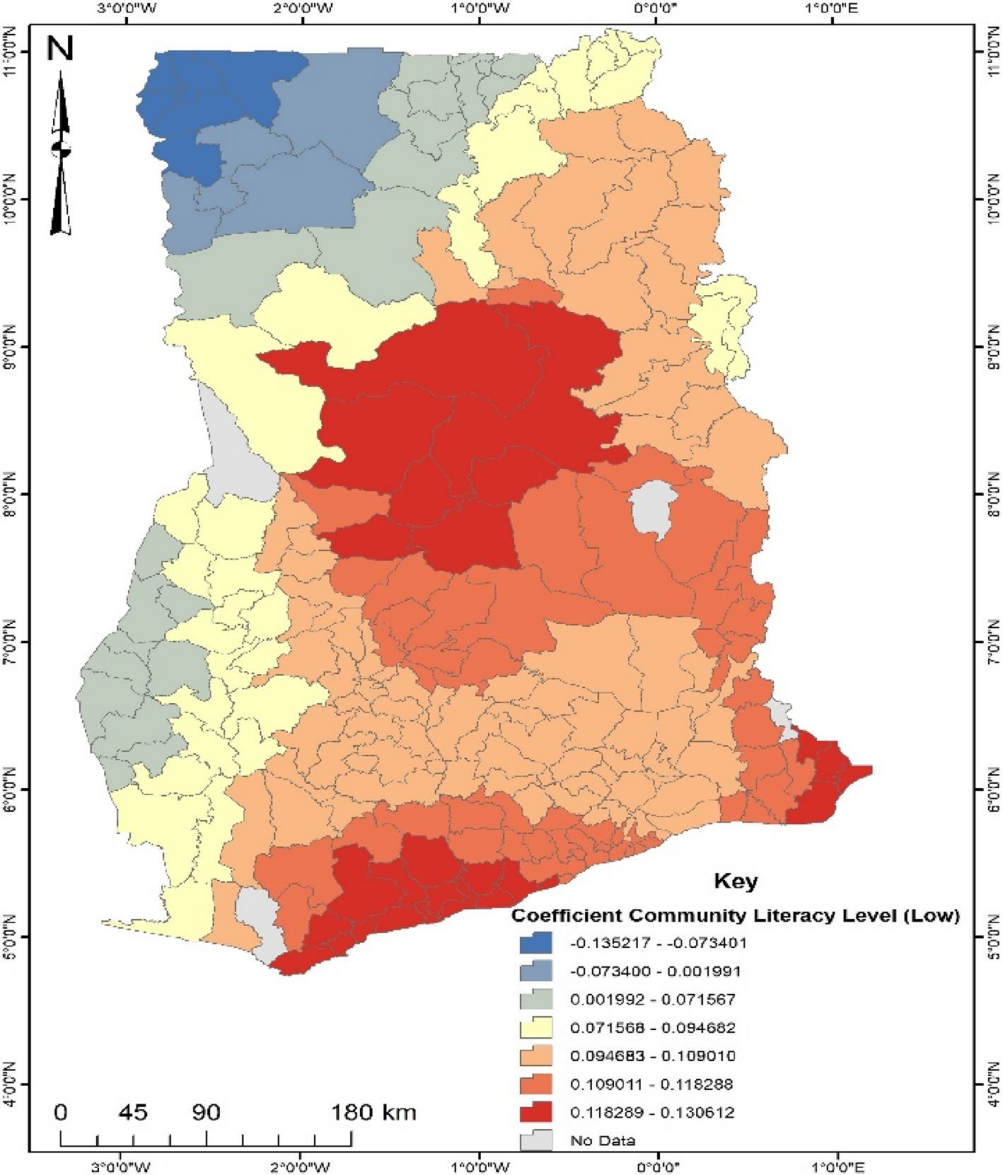
Coefficient of Literacy Level (Low). Source: Computed from 2017 Maternal Health Survey

According to Fig. 7, about 9–13% of women living in the south-eastern and east of the middle belt were more likely to be noncompliant with ANC due to low community literacy level. This means that women with low community literacy level have a higher tendency to engage in noncompliance of ANC in the aforementioned areas of Ghana. However, women in the north-west are less likely (1–13%) to engage in noncompliance of ANC regardless of their low community literacy level.

## Discussion

In this study, we explored the geospatial differentials in women’s noncompliance with WHO’s recommended ANC 8-contact model in Ghana using data from the 2017 Ghana Maternal Health Survey (GMHS). In consonance with existing literature, the preliminary analysis revealed that a number of socio-demographic characteristics of women align with noncompliance with the WHO’s recommended ANC 8-contact model. Age, wealth status, educational level, marital status, parity, mass media, community socioeconomic status and place of residence had a significant relationship with ANC noncompliance among Ghanaian women. In Nigeria, where Aliyu et al. [25] reported that 27%, 62% and 12% of women started ANC in the first, second and third trimesters respectively; the leading factors for this relatively low ANC coverage included maternal education, region and place of residence of the women [25].

In the case of El-Khatib et al. [3] where 46% of the women in Nigeria fell below the WHO recommended ANC 8-contact model, this was ascribed to the type of residency, educational level, household size, use of contraceptives together with the distance covered to access healthcare [3]. Another cross-sectional study from Jordan reports that maternal education and other socio-demographic factors affect women’s ability to comply with the WHO recommended ANC 8-contact model [26]. Juxtaposing our finding with reports from earlier studies point to the need for some crucial reforms and national priorities as far as maternal health and wellbeing are concerned. Thus, education and intensified primary healthcare models could be some of the critical interventions required to enhance ANC compliance and overall improvement in service utilization and maternal health.

Women without the National Health Insurance Scheme in the middle belt were more likely to be non-compliant with ANC. The instrumental role of the National Health Insurance Scheme in maternal healthcare delivery has been documented extensively within the context of Ghana [27, 28], West Africa [29] and sub-Saharan Africa at large [30]. The findings, coupled with the consistent reports from previous studies from Ghana and across the sub-Sahara African sub-region suggest that it is instructive for the government of Ghana to ensure the sustainability of the NHIS. It will be prudent to implement health education programmes to educate all women in the reproductive age to subscribe to the NHIS in order to accelerate Ghana’s prospects of achieving the first and third targets of the third Sustainable Development Goal (SDG). Target one of the third SDG enjoins all countries to attain less than 70 per 100,000 Maternal Mortality Ratio (MMR) whilst target two urges all countries to end preventable death of newborns and reduce neonatal mortality to at least 12 per 1,000 live births by 2030 [31].

Relative to married women, it was noted that women who had never been in union had higher odds of ANC noncompliance. This is in conformity with the literature that have revealed that married women have increased prospects of completing ANC schedule. For instance, in Indonesia, Wulandari and colleagues realized that married women’s had 1.8 times propensity of completing ANC compared to women who were not in union [32]. Similar findings have emerged from Senegal [33]. The seemingly relative advantage of married women over unmarried women may be contextualized in the agency of marriage, whereby male partners remind and support their wives to complete ANC regimen. Some evidences have shown that male partners are sometimes actively involved in ANC [34, 35]. Unmarried women may be devoid of such relative advantage, therefore being less motivated to complete ANC.

Notable hotspots for noncompliance of ANC predominantly spanned across north-eastern Ghana. Women in northern Ghana face diverse challenges in healthcare access. This may be attributable to both institutional and non-institutional (community/individual) level factors. For instance, it is common knowledge in Ghana that most essential health care providers refuse postings to the northern parts of the country, due to the unparalleled development and harsh climate conditions relative to the south [36]. Statistics from the Ministry of Health indicate that the Northern region, for instance, has 2% (38) of the medical officers and 7.4% (285) of the midwives for the 10.2% of Ghana’s population inhabiting the region. By contrast, a southern based region like Volta has 2.7% (43) of the medical officers and 10.1% (386) of the midwives catering for the 8.6% of Ghana’s population living in that region [37]. This disparity compromises women’s access to health staff at regular intervals and within reasonable distances, where they can access with ease.

Although not significant in multivariate modelling, spatial analysis at the community level indicated that low literacy levels might contribute to noncompliance in the southeast and east of the middle belt. As demonstrated in this study higher education levels, associated with literacy, impact noncompliance. Health literacy, or an individual’s ability to find, understand, and use healthcare, also has the ability to influence care-seeking behaviours of an individual as well as those around them [38]. New models of ANC care, such as group ANC care, have demonstrated higher rates of health literacy among participants [39] as well as the potential to improve communication and community among participants [40]. As healthcare providers are in limited supply, particularly in rural regions of northern Ghana, it is essential to consider alternative models of care that may improve both efficiency and quality such increasing the Community based health planning services (CHPS).

At the community/individual level, poverty is a major setback to women’s ability to frequent health facilities for ANC in the eastern part of Ghana. It was also evident that women with low community socioeconomic status were more likely to be non-compliant with ANC. Consistent evidence clearly indicates that compared to the south, poverty dominates in northern Ghana [41, 42]. The decision to utilise healthcare or not is a difficult one to make when the person lacks transport fare and the financial capacity to offset her healthcare bills. It is worth noting, there are some additional costs borne by women who are even covered by the National Health Insurance Scheme, such as costs associated with some laboratory examinations during pregnancy as well as items for the labor and delivery such as disinfectants, soap, rubber pads, and baby clothes [43]. As pioneered by Thaddeus and Maine [44], poverty may trigger the first delay, which occurs at the decision-making stage regarding whether it is worthy to access healthcare in the first place.

### Strengths and limitations of the study

This study utilises recent national survey data to uniquely visualise locations where noncompliance with WHO’s recommendation for ANC attendance exists in Ghana, using geospatial analysis. The geospatial analysis provided important regionally specific information that could be used to tailor interventions and policies by region as opposed to enacting blanket policies and interventions across countries. This methodology has the potential to provide important insight to other global health issues. Appropriate methodological procedures and rigorous analytical procedures were employed at all levels of analysis. Meanwhile, readers should be mindful of the following limitations when interpreting the results. The study is cross-sectional in nature and therefore it is not possible to draw causal inferences between the explanatory variables and noncompliance with ANC.

## Conclusion

The study focused on geospatial differentials in women’s noncompliance with the WHO’s recommended ANC attendance in Ghana. It was evident from the logistic regression results that age, wealth status, educational level, marital status, parity, mass media, community socioeconomic status and place of residence drive ANC noncompliance among Ghanaian women. Women in north-eastern Ghana, those in the middle belt without the National Health Insurance Scheme, and women with low community socioeconomic status were noted to be noncompliant with ANC as recommended by the WHO. The study has shown that in order to achieve targets one and three of SDG 3, the government of Ghana, the Ministry of Health together with the Ghana Health Service may have to intensify health education in the identified areas to project the importance of adhering to the recommendations of the WHO regarding ANC. Media advertisements, equitable distribution of health professionals across the country may help reduce the noncompliance rate in Ghana.

## Supplementary Information

**Additional file 1: Appendix A.** Spatial autocorrection of ANC. **Appendix B.** OLS results of spatial predictors of Non-compliance of ANC.

## Data Availability

The datasets generated and/or analysed during the current study are available in the Measure DHS repository, https://dhsprogram.com/data/available-datasets.cfm. Or The DHS Program—Available Datasets.

## Abbreviations

ANC: Antenatal Care
CI: Confidence interval
GMHS: Ghana Maternal Health Survey
LR: Likelihood ratio
OR: Odds ratios
SDG: Sustainable Development Goal
