# Supplementary material for: Non-adherence to WHO’s recommended 8-contact model: geospatial analysis of the 2017 Maternal Health Survey
Source: BMC Pregnancy Childbirth. 2023 Mar 18;23:192. doi: 10.1186/s12884-023-05504-w (PMC10024456; doi:10.1186/s12884-023-05504-w)
Supplement: Supplementary file 1 — Additional file 1: Appendix A. Spatial autocorrection of ANC. Appendix B. OLS results of spatial predictors of Non-compliance of ANC. [file 12884_2023_5504_MOESM1_ESM.docx]

**Appendices**


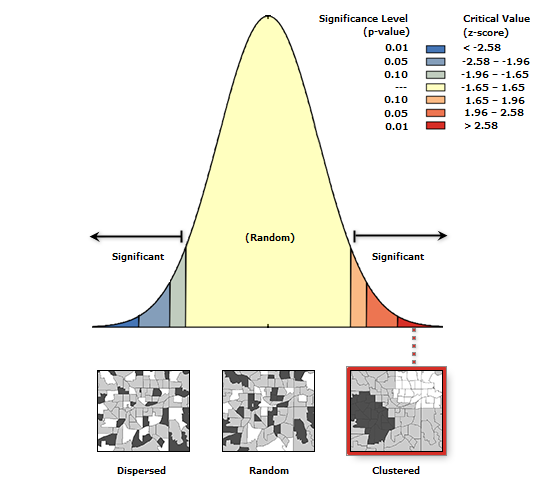


Appendix A: Spatial autocorrection of ANC

Source: Computed from 2017 Maternal Health Survey

Appendix B: OLS results of spatial predictors of Non-compliance of ANC

| **Variable** | **Coefficient [a]** | **StdError** | **t-Statistic** | **Probability [b]** | **VIF [c]** |
| --- | --- | --- | --- | --- | --- |
| Intercept | 0.267103 | 0.08663 | 3.08324 | 0.002348* | -------- |
| AGE (15-19YRS) | 0.071533 | 0.152863 | 0.467958 | 0.640339 | 1.430229 |
| TYPE OF RESIDENCE (RURAL) | -0.053581 | 0.043538 | -1.230678 | 0.219905 | 1.815599 |
| EDUCATION (NO FORMAL) | 0.078338 | 0.073119 | 1.071365 | 0.285303 | 3.333023 |
| SEX (FEMALE) | 0.05841 | 0.077705 | 0.751686 | 0.453125 | 1.987375 |
| WEALTH STATUS (POOREST) | -0.010851 | 0.071849 | -0.15102 | 0.880106 | 4.574258 |
| NO MEDICAL HELP | 0.116105 | 0.062099 | 1.869672 | 0.063007 | 1.984728 |
| NO NHIS | 0.179321 | 0.055435 | 3.234816 | 0.001437* | 1.166874 |
| MARITAL (NEVER IN A UNION) | -0.295219 | 0.137436 | -2.148054 | 0.032915* | 1.863012 |
| MASS MEDIA (NO) | 0.135855 | 0.098566 | 1.378324 | 0.169668 | 2.429664 |
| PARITY | 0.162158 | 0.088051 | 1.84163 | 0.067028 | 1.64158 |
| HOUSEHOLD (<5) | -0.132643 | 0.064937 | -2.042642 | 0.042407* | 1.584408 |
| COMMUNITY SOCIOECONOMIC STATUS (LOW) | 0.215481 | 0.041755 | 5.160645 | 0.000001* | 2.247652 |
| COMMUNITY EDUCATION STATUS | 0.099936 | 0.040849 | 2.446459 | 0.015289* | 2.137323 |

Source: Computed from 2017 Maternal Health Survey
